# Supplementary material for: Imaging of Peritoneal Metastases in Ovarian Cancer Using MDCT, MRI, and FDG PET/CT: A Systematic Review and Meta-Analysis
Source: Cancers (Basel). 2024 Apr 11;16(8):1467. doi: 10.3390/cancers16081467 (PMC11048266; doi:10.3390/cancers16081467)
Supplement: Supplementary file 1 [file cancers-16-01467-s001.zip › cancers-2887393-supplementary.pdf]

cancers

MDPI

Table S1. Results of the Per-Patient analysis for MDCT (TP: true-positive, FN: false-negative; FP: false-positive; TN: true-negative; PPV: positive-predictive value; NPV: negative-predictive value; MPR: multiplanar reformation).

Diagnostic values of MDCT on a Per-Patient Basis									
**Study**	**TP**	**FN**	**FP**	**TN**	**Sensitivity (%)**	**Specificity (%)**	**PPV (%)**	**NPV (%)**	**Accuracy (%)**
**Pannu et al [52]**									
axial CT + MPR-arterial phase, infracolic omentum, observer 2	7	0	0	10	100	100	100	100	100
**Hynninen et al [63]**									
left 'high risk upper abdomen'	8	6	0	8	57	100	100	57	72
**Kim et al [64]**									
	23	3	7	13	88.5	65	76.7	81.3	78.3
**Mazzei et al [65]**									
	38	0	3	2	100	40	93	100	93
**Nasser et al [26]**									
porta hepatis	4	3	1	147	57	99	80	98	97.4
**Rodolfino et al [69]**									
experienced readers, portal + delayed phase	27	2	3	8	93.1	72.7	90	80	87.5
**Tawakol et al [70]**									
	52	23	9	52	69	85	85.2	69.3	76
**Cerci et al [71]**									
peritoneal carcinomatosis	35	26	4	47	57.4	92.2	89.7	64.4	73.2
**Bagul et al [72]**									
diffuse peritoneal thickening	17	0	0	19	100	100	100	100	100
**Michielsen et al [73]**									
duodenum, stomach, celiac trunk carcinomatosis	11	5	1	77	68.8	98.7	91.7	93.9	93.6
**Rajan et al [74]**									
left upper	1	0	1	38	100	97.4	50	100	97.5
**Alcazar et al [75]**									
major omentum	35	11	3	44	76.1	93.6	92.1	80	85
**An et al [27]**									
	47	4	3	4	92.16	57.14	94	50	87.93
**Mikkelsen et al [30]**									
liver/duodenum/ pancreas/gastric ventricle	3	4	0	43	43	100	100	92	92
**Fischerova et al [78]**									
	41	2	10	14	95	58	80	88	82

Table S2. Results of the Per-Patient analysis for MRI (TP: true-positive, FN: false-negative; FP: false-positive; TN: true-negative; PPV: positive-predictive value; NPV: negative-predictive value; PMs: peritoneal metastases).

Diagnostic values of MRI on a Per-Patient Basis									
**Study**	**TP**	**FN**	**FP**	**TN**	**Sensitivity (%)**	**Specificity (%)**	**PPV (%)**	**NPV (%)**	**Accuracy (%)**
**Tempany et al [51]**									
	39	2	27	107	95	80	59.1	98.2	83.4
**Ricke et al [53]**									
greater omentum	*8*	*13*	2	34	*38.1*	*94.4*	*80*	*72.3*	73.7
**Sanli et al [61]**									
PMs: 2-3 cm	15	1	0	31	93.3	100	100	96.9	97.8
**Espada et al [62]**									
omental extension: spleen parenchyma, splenic hilum, stomach, lesser sac	11	0	0	23	100	100	100	100	100
**Michielsen et al [73]**									
duodenum, stomach, celiac trunk	15	1	1	77	93.8	98.7	93.8	98.7	97.9
**Mikkelsen et al [30]**									
celiac trunk/superior mesenteric artery/bowel mesentery root	6	8	0	33	40	100	100	79	83
**Fischerova et al [78]**									
	40	3	5	19	93	79	89	86	88

Table S3. Results of the Per-Patient analysis for FDG PET/CT (TP: true-positive, FN: false-negative; FP: false-positive; TN: true-negative; PPV: positive-predictive value; NPV: negative-predictive value).

Diagnostic values of FDG PET/CT on a Per-Patient Basis									
**Study**	**TP**	**FN**	**FP**	**TN**	**Sensitivity (%)**	**Specificity (%)**	**PPV (%)**	**NPV (%)**	**Accuracy (%)**
**Pannu et al [54]**									
	8	3	3	2	72.7	40	72.7	40	62.5
**Sanli et al [61]**									
PMs: 2-3 cm	15	0	0	32	100	100	100	100	100
**Hynninen et al [63]**									
omentum	31	3	1	6	91	86	97	67	90
**Kim et al [64]**									
	25	1	2	18	96.2	90	92.6	94.7	93.5
**Tawakol et al [70]**									
	72	3	0	61	96	100	100	95.3	98
**Tsoi et al [77]**									
	25	2	4	18	92.6	81.8	86.2	90	87.8
**Mikkelsen et al [30]**									
liver/duodenum/ pancreas/gastric ventricle	6	1	1	42	85	98	86	98	96

**Mallet et al [44]**									
AR-0 *(central)*	69	4	6	5	94.5	45.5	92	55.5	88.1

Table S4. Results of the Per-Region analysis for MDCT (TP: true-positive, FN: false-negative; FP: false-positive; TN: true-negative; PPV: positive-predictive value; NPV: negative-predictive value).

**Diagnostic values of MDCT on a Per-Region Basis**									
**Study**	**TP**	**FN**	**FP**	**TN**	**Sensitivity (%)**	**Specificity (%)**	**PPV (%)**	**NPV (%)**	**Accuracy (%)**
**Kitajima et al [56]**									
	27	18	4	83	60	95.4	87.1	82.2	83.3
**Kitajima et al [57]**									
liver surface	2	0	0	38	100	100	100	100	100
**Choi et al [58]**									
	114	137	135	355	45	72	46	72	63.3
**Metser et al [59]**									
	336	78	27	1404	81.2	98.1	92.6	94.7	94.3
**Hynninen et al [63]**									
	196	288	20	216	41	92	90.7	42.8	57
**Mazzei et al [65]**									
	141	54	73	291	72	80	66	84	77
**Michielsen et al [66]**									
	136	72	47	220	65	82	74	75	75
**Schmidt et al [67]**									
	71	3	5	56	96	92	95	94	95
**Lopez-Lopez et al [68]**									
	79	199	9	480	35	98	90	72	73
**Rodolfino et al [69]**									
experienced readers, portal + delayed phase	171	11	21	304	94	93.5	89.1	96.5	93.7
**Abdalla Ahmed et al [76]**									
	925	50	20	130	94.9	86.7	97.9	72.2	93.8
**An et al [27]**									
	184	108	39	108	63.01	73.47	82.51	50	66.51

Table S5. Results of the Per-Region analysis for MRI (TP: true-positive, FN: false-negative; FP: false-positive; TN: true-negative; PPV: positive-predictive value; NPV: negative-predictive value).

**Diagnostic values of MRI on a Per-Region Basis**									
**Study**	**TP**	**FN**	**FP**	**TN**	**Sensitivity (%)**	**Specificity (%)**	**PPV (%)**	**NPV (%)**	**Accuracy (%)**
**Kim et al [55]**									
	12	2	0	22	86	100	100	92	94
**Michielsen et al [66]**									
	189	19	24	243	91	91	89	93	91
**Schmidt et al [67]**									
	73	1	10	51	98	84	91	96	93

Table S6. Results of the Per-Region analysis for FDG PET/CT (TP: true-positive, FN: false-negative; FP: false-positive; TN: true-negative; PPV: positive-predictive value; NPV: negative-predictive value).

Diagnostic values of FDG PET/CT on a Per-Region Basis									
**Study**	**TP**	**FN**	**FP**	**TN**	**Sensitivity (%)**	**Specificity (%)**	**PPV (%)**	**NPV (%)**	**Accuracy (%)**
**Pannu et al [54]**									
	7	24	3	16	22.5	84.2	70	40	46
**Kim et al [55]**									
	6	8	1	21	43	96	86	72	75
**Kitajima et al [56]**									
CECT	36	9	2	85	80	97.7	94.7	90.4	91.7
**Kitajima et al [57]**									
liver surface	2	0	0	38	100	100	100	100	100
**De laco et al [60]**									
	243	65	12	26	78.9	68.4	95.3	28.5	77.7
**Hynninen et al [63]**									
	237	226	25	205	51	89	90.4	47.5	64
**Michielsen et al [66]**									
	108	100	39	228	52	85	73	70	71
**Schmidt et al [67]**									
	70	4	2	59	95	96	98	92	96
**Lopez-Lopez et al [68]**									
	69	209	34	455	24	93	66	68	68
**Tsoi et al [77]**									
	44	14	4	673	75.9	99.4	91.7	98	97.6
**Feng et al [42]**									
	208	78	41	231	72.7	84.9	83.5	74.8	78.5

Table S7. Results of the Per-Patient analysis for MDCT in different abdominopelvic regions (TP: true-positive, FN: false-negative; FP: false-positive; TN: true-negative; PPV: positive-predictive value; NPV: negative-predictive value; ARs: abdominopelvic regions; MPR: multiplanar reformation).

Diagnostic values of MDCT on a Per-Patient Basis in different ARs									
**Study**	**TP**	**FN**	**FP**	**TN**	**Sensitivity (%)**	**Specificity (%)**	**PPV (%)**	**NPV (%)**	**Accuracy (%)**
AR0									
**Pannu et al [52]** *axial CT + MPR-arterial phase, infracolic omentum, observer 2*	7	0	0	10	100	100	100	100	100
**Hynninen et al [63]** *omentum*	27	7	2	5	79	71	93	42	78
**Cerci et al [71]** *omentum*	36	17	3	55	67.9	94.8	92.3	76.4	82
**Bagul et al [72]** *omentum*	34	1	0	1	97.1	100	100	50	97.2
**Rajan et al [74]**	14	10	2	14	58.3	87.5	87.5	58.3	70

central									
**Alcazar et al [75]** *major omentum*	35	11	3	44	76.1	93.6	92.1	80	85
**Fischerova et al [78]** *greater omentum*	40	2	9	16	95	64	82	89	84
AR1									
**Pannu et al [52]** *axial CT + MPR-arterial phase, porta hepatis/gallbladder, observer 2*	2	2	0	13	50	100	100	86.6	88.2
**Hynninen et al [63]** *right 'high risk upper abdomen'*	17	14	0	5	55	100	100	26	61
**Nasser et al [26]** *porta hepatis*	4	3	1	147	57	99	80	98	97.4
**Bagul et al [72]** *right subdiaphragm*	32	3	0	1	91.4	100	100	25	91.6
**Rajan et al [74]** *right upper*	1	3	5	31	25	86.1	16.6	91.1	80
**Alcazar et al [75]** *hepatic hilum*	2	8	0	83	20	100	100	91.2	91.4
**Mikkelsen et al [30]** *porta hepatis/ hepatoduodenal ligament*	6	11	1	32	35	97	86	74	76
**Fischerova et al [78]** *right diaphragm*	26	10	3	28	72	90	90	74	81
AR2									
**Pannu et al [52]** *axial CT + MPR-arterial phase, lesser sac, observer 2*	2	1	3	11	66.6	78.5	40	91.6	76.4
**Bagul et al [72]** *lesser sac*	8	7	0	21	53.3	100	100	75	80.5
**Rajan et al [74]** *epigastrium*	3	3	3	31	50	91.1	50	91.1	85
**Fischerova et al [78]** *lesser omentum*	4	4	3	53	50	95	57	93	89
AR3									
**Pannu et al [52]**	2	0	1	14	100	93.3	66.6	100	94.1

axial CT + MPR-arterial phase, spleen, observer 2									
**Hynninen et al [63]** *left 'high risk upper abdomen'*	8	6	0	8	57	100	100	57	72
**Nasser et al [26]** *spleen*	5	14	2	134	26	99	71	91	90
**Bagul et al [72]** *left subdiaphragm*	22	5	0	9	81.4	100	100	35.7	81.6
**Rajan et al [74]** *left upper*	1	0	1	38	100	97.4	50	100	97.5
**Alcazar et al [75]** *spleen*	2	3	0	88	40	100	100	96.7	97
**Fischerova et al [78]** *left diaphragm*	15	6	3	42	71	93	83	88	86
AR4									
**Bagul et al [72]** *left paracolic region*	19	8	0	9	70.3	100	100	53.7	77.7
**Rajan et al [74]** *left flank*	7	0	3	30	100	90.9	70	100	92.5
**Fischerova et al [78]** *left paracolic gutter*	20	9	8	30	69	79	71	77	75
AR5-7									
**Pannu et al [52]** *axial CT + MPR-arterial phase, pelvis, observer 1*	8	4	0	5	66.6	100	100	55.5	76.4
**Hynninen et al [63]** *sigmoid serosa*	5	20	0	9	20	100	100	31	41
**Nasser et al [26]** *rectum*	15	24	1	115	39	99	94	83	84
**Cerci et al [71]** *perivesical-perirectal fat*	23	31	2	53	42.6	96.4	92	63.1	62.7
**Bagul et al [72]** *uterus and ovary*	34	0	0	2	100	100	100	100	100
**Rajan et al [74]** *pelvis*	34	2	1	3	94.4	75	97.1	60	92.5
**Alcazar et al [75]** *pelvic peritoneum*	35	24	3	31	59.3	91.2	92.1	56.4	71
**Fischerova et al [78]** pelvis	42	5	5	14	89	74	89	74	85
AR6									
**Pannu et al [52]**	4	1	0	12	80	100	100	92.3	94.1

axial CT + MPR-arterial phase, ovarian mass, observer 2									
**Nasser et al [26]** *rectum*	15	24	1	115	39	99	94	83	84
**Cerci et al [71]** *perivesical-perirectal fat*	23	31	2	53	42.6	96.4	92	63.1	62.7
**Bagul et al [72]** *uterus and ovary*	34	0	0	2	100	100	100	100	100
**Rajan et al [74]** *pelvis*	34	2	1	3	94.4	75	97.1	60	92.5
**Alcazar et al [75]** *rectosigmoid*	9	18	7	59	33.3	89.4	56.2	76.6	73
**Fischerova et al [78]** *rectosigmoid*	34	2	8	21	94	72	81	91	85
AR8									
**Bagul et al [72]** *right paracolic region*	22	11	0	3	68.5	100	100	23	71.4
**Rajan et al [74]** *right flank*	7	1	1	31	87.5	96.8	87.5	96.8	95
**Fischerova et al [78]** *right paracolic gutter*	20	8	8	31	71	79	71	79	76
diaphragm									
**Pannu et al [52]** *axial CT + MPR-arterial phase, observer 2*	5	6	0	6	45.5	100	100	50	64.7
**Hynninen et al [63]**	16	18	0	6	47	100	100	25	55
**Nasser et al [26]**	19	42	1	93	32	99	95	70	72.3
**Cerci et al [71]**	0	20	1	80	0	98.8	0	80	79.2
**Bagul et al [72]** *right subdiaphragm*	32	3	0	1	91.4	100	100	25	91.6
**Fischerova et al [78]** right, left diaphragm	26	10	3	28	72	90	90	74	81
small bowel									
**Hynninen et al [63]** *small bowel serosa*	2	12	2	25	14	93	50	68	66
**Mazzei et al [65]**	7	5	4	27	58	87	64	84	79

**Nasser et al [26]**	7	10	1	137	44	99	88	94	92.9
**Bagul et al [72]** *small bowel serosa*	8	12	1	15	35	93.7	87.5	53.5	61.1
**Rajan et al [74]** *lower ileum*	3	2	0	35	60	100	100	94.5	95
**Alcazar et al [75]**	7	5	7	74	58.3	91.4	50	93.7	87.1
**Fischerova et al [78]** *small bowel serosa*	11	8	4	42	58	91	73	84	82
colon									
**Hynninen et al [63]** *large bowel serosa*	13	53	2	40	20	95	87	37	49
**Nasser et al [26]** *large bowel*	17	20	2	116	46	98	89	85	86
**Bagul et al [72]** *large bowel serosa*	12	17	0	7	41.3	100	100	29.1	52.7
**Fischerova et al [78]** *large bowel serosa*	4	15	5	43	21	90	44	74	70
mesentery									
**Pannu et al [52]** *axial CT + MPR-arterial phase, mesenteric root, observer 1*	1	2	0	14	33.3	100	100	87.5	88.2
**Hynninen et al [63]** *small bowel mesentery*	7	19	0	13	27	100	100	41	51
**Nasser et al [26]**	12	28	1	114	31	99	92	81	81.3
**Cerci et al [71]**	12	37	4	48	24.5	92.3	75	56.5	59.4
**Bagul et al [72]** *small bowel mesentery*	11	10	0	15	52.3	100	100	60	72.2
**Michielsen et al [73]** *superior mesenteric artery, mesenteric root*	3	5	0	86	37.5	100	100	94.5	94.7
**Alcazar et al [75]**	1	3	7	82	25	92.1	14.3	95.3	89.2
**Mikkelsen et al [30]** *celiac trunk/superior mesenteric artery/bowel mesentery root*	2	12	0	33	14	100	100	73	74.5

**Fischerova et al [78]** *small and large bowel mesentery*	17	13	2	35	57	95	89	73	78

Table S8. Results of the Per-Patient analysis for MRI in different abdominopelvic regions (TP: true-positive, FN: false-negative; FP: false-positive; TN: true-negative; PPV: positive-predictive value; NPV: negative-predictive value).

**Diagnostic values of MRI on a Per-Patient Basis in different ARs**									
**Study**	**TP**	**FN**	**FP**	**TN**	**Sensitivity (%)**	**Specificity (%)**	**PPV (%)**	**NPV (%)**	**Accuracy (%)**
					AR0				
**Tempany et al [51]** *omentum*	40	30	82	128	57.1	61	32.8	81	60
**Ricke et al [53]** *greater omentum*	8	13	2	34	38.1	94.4	80	72.3	73.7
**Fischerova et al [78]** *greater omentum*	38	4	5	20	90	80	88	83	87
					diaphragm				
**Tempany et al [51]** *subdiaphragmatic spaces*	27	18	106	129	60	55	20.3	87.8	55.7
**Ricke et al [53]**	11	4	5	37	73.3	88.1	68.8	90.2	84.2
**Espada et al [62]**	4	1	1	28	80	96.6	80	96.6	94
**Fischerova et al [78]** *right, left diaphragm*	26	10	1	30	72	97	96	75	84
					mesentery				
**Tempany et al [51]**	20	18	94	148	52.6	61.1	17.5	89.2	60
**Espada et al [62]** *small and/or large bowel mesentery*	8	0	4	22	100	84.6	66.6	100	88.2
**Michielsen et al [73]** *superior mesenteric artery, mesenteric root*	8	0	1	85	100	98.8	88.9	100	98.9
**Mikkelsen et al [30]** *celiac trunk/superior mesenteric artery/bowel mesentery root*	6	8	0	33	40	100	100	79	83
**Fischerova et al [78]** *small and large bowel mesentery*	16	14	4	33	53	89	80	70	73

Table S9. Results of the Per-Patient analysis for FDG PET/CT in different abdominopelvic regions (TP: true-positive, FN: false-negative; FP: false-positive; TN: true-negative; PPV: positive-predictive value; NPV: negative-predictive value).

Diagnostic values of FDG PET/CT on a Per-Patient Basis in different ARs									
**Study**	**TP**	**FN**	**FP**	**TN**	**Sensitivity (%)**	**Specificity (%)**	**PPV (%)**	**NPV (%)**	**Accuracy (%)**
AR0									
**Hynninen et al [63]** *omentum*	31	3	1	6	91	86	97	67	90
**Tsoi et al [77]** *omentum*	5	1	2	41	83.3	95.3	71.4	97.6	93.9
**Mallet et al [44]** *central*	69	4	6	5	94.5	45.5	92	55.5	88.1
AR1									
**Hynninen et al [63]** *right 'high risk upper abdomen'*	20	11	0	5	65	100	100	31	70
**Tsoi et al [77]** *right subhepatic space*	2	0	0	47	100	100	100	100	100
**Mikkelsen et al [30]** *porta hepatis/ hepatoduodenal ligament*	11	6	3	30	65	91	79	83	82
**Mallet et al [44]** *right upper*	53	14	5	11	79.1	68.8	91.4	44	77.1
AR3									
**Hynninen et al [63]** *left 'high risk upper abdomen'*	3	11	2	6	21	75	60	35	41
**Tsoi et al [77]** *gastric serosa*	1	0	0	48	100	100	100	100	100
**Mallet et al [44]** *left upper*	46	10	9	19	82.1	67.9	83.6	65.5	77.4
AR5-7									
**Hynninen et al [63]** *sigmoid*	19	7	2	8	73	80	91	53	75
**Tsoi et al [77]** *pelvis*	14	0	1	34	100	97.1	93.3	100	98
**Mallet et al [44]** *pelvis*	75	3	3	0	96.2	0	96.2	0	92.6
mesentery									
**Hynninen et al [63]** *small bowel mesentery*	16	9	1	12	64	92	94	57	74

**Tsoi et al [77]**	2	3	0	44	40	100	100	93.6	93.9
**Mikkelsen et al [30]** *celiac trunk/superior mesenteric artery/bowel mesentery root*	2	12	0	33	14	100	100	73	74.5

Table S10. Results of the Per-Region analysis for MDCT in different abdominopelvic regions (TP: true-positive, FN: false-negative; FP: false-positive; TN: true-negative; PPV: positive-predictive value; NPV: negative-predictive value; ARs: abdominopelvic regions).

**Diagnostic values of MDCT on a Per-Region Basis in different ARs**									
**Study**	**TP**	**FN**	**FP**	**TN**	**Sensitivity (%)**	**Specificity (%)**	**PPV (%)**	**NPV (%)**	**Accuracy (%)**
AR0									
**Kitajima et al [57]** *omentum*	3	6	1	30	33	97	75	83.3	83
**Choi et al [58]** *omentum*	6	14	15	22	30	59	29	61	49.1
**Michielsen et al [66]** *omentum*	18	5	3	5	78	63	86	50	74
**An et al [27]** *omentum*	27	10	1	10	72.97	90.91	96.43	50	77.08
AR5-7									
**Kitajima et al [57]** *cul de sac*	2	6	1	31	25	97	66.6	83.8	75
**Choi et al [58]** *bladder dome area*	0	16	2	39	0	95	0	70	68.4
**Michielsen et al [66]** *left pelvic*	17	4	3	7	81	70	85	64	77
**An et al [27]** *pelvic peritoneum*	22	21	7	25	51.16	78.12	75.86	54.35	62.67
AR6									
**Kitajima et al [57]** *cul de sac*	2	6	1	31	25	97	66.6	83.8	75
**Choi et al [58]** *bladder dome area*	0	16	2	39	0	95	0	70	68.4
**Michielsen et al [66]** *bladder surface*	8	9	0	11	47	100	100	55	68
diaphragm									
**Kitajima et al [57]**	0	1	0	39	0	100	-	97.5	98
**Choi et al [58]** left subdiaphragmatic area	16	18	2	21	47	91	89	54	65
**Michielsen et al [66]** *left diaphragm*	7	2	4	15	78	79	64	88	79
**An et al [27]**	1	15	1	7	6.25	87.50	50	31.82	33.33

subdiaphragmatic space									
					**mesentery**				
**Kitajima et al [57]**	2	5	1	32	29	97	66.7	86.4	85
**Choi et al [58]** *small bowel mesentery*	3	11	3	40	21	93	50	78	75.4
**Michielsen et al [66]** *small bowel mesentery*	8	4	1	16	67	94	89	80	83
**An et al [27]**	10	10	5	10	50	66.67	66.67	50	57.14

Table S11. Results of the Per-Region analysis for FDG PET-CT in different abdominopelvic regions (TP: true-positive, FN: false-negative; FP: false-positive; TN: true-negative; PPV: positive-predictive value; NPV: negative-predictive value; ARs: abdominopelvic regions).

**Diagnostic values of FDG PET/CT on a Per-Region Basis in different ARs**									
**Study**	**TP**	**FN**	**FP**	**TN**	**Sensitivity (%)**	**Specificity (%)**	**PPV (%)**	**NPV (%)**	**Accuracy (%)**
					AR0				
**Kitajima et al [57]** *omentum*	5	4	1	30	56	97	83.3	88.2	88
**De laco et al [60]** *mesogastrium*	35	2	2	1	94.6	33.3	94.6	33.3	90
**Michielsen et al [66]** *omentum*	18	5	1	7	78	88	95	58	81
**Feng et al [42]** *central*	24	5	2	12	82.8	85.7	92.3	70.6	83.7
					AR1				
**Pannu et al [54]** *right upper quadrant*	0	1	0	3	0	100	-	75	75
**De laco et al [60]** *right upper*	21	13	0	2	61.7	100	100	13.3	64
**Michielsen et al [66]** *subhepatic/ Morrison's space*	5	5	9	32	50	78	36	86	73
**Feng et al [42]** *right upper*	24	7	1	11	77.4	91.7	96	61.1	81.4
					AR3				
**Pannu et al [54]** *left upper quadrant*	1	1	1	1	50	50	50	50	50
**De laco et al [60]** *left upper*	18	10	1	6	64.3	85.7	94.7	37.5	68.6
**Michielsen et al [66]** *splenic surface*	1	0	3	18	100	86	25	100	86
**Feng et al [42]** *left upper*	15	1	5	22	93.8	81.5	75	95.7	86

				AR4					
**De laco et al [60]** *left flank*	29	6	2	2	82.8	50	93.5	25	79.5
**Michielsen et al [66]** *left lateroconal*	11	5	2	11	69	85	85	69	76
**Feng et al [42]** *left flank*	13	6	4	20	68.4	83.3	76.5	76.9	76.7
				AR5-7					
**Pannu et al [54]**	4	13	0	4	23.5	100	100	23.5	38.1
**Kitajima et al [57]** *cul de sac*	4	4	1	31	50	97	80	88.6	80
**De laco et al [60]** *left lower*	26	10	2	2	72.2	50	92.8	16.6	70
**Michielsen et al [66]** *Douglas pouch*	9	10	0	10	47	100	100	50	66
**Feng et al [42]** *left lower*	21	11	2	9	65.6	81.8	91.3	45	69.8
				AR8					
**De laco et al [60]** *right flank*	29	5	1	4	85.3	80	96.6	44.4	84.6
**Michielsen et al [66]** *right lateroconal*	11	4	3	10	73	77	79	71	75
**Feng et al [42]** *right flank*	14	7	3	19	66.7	86.4	82.4	73.1	76.7
